# Privatisation of government services in Australia: what is known about health and equity impacts

**DOI:** 10.1186/s12992-024-01036-w

**Published:** 2024-04-16

**Authors:** Julia Anaf, Toby Freeman, Fran Baum

**Affiliations:** Stretton Health Equity, Stretton Institute, North Tce Campus, University of Adelaide, 5005 Adelaide, Australia

**Keywords:** Privatisation, Outsourcing, Contracting out, Private public partnerships, Health, Health equity, Social determinants of health, Commercial determinants of health

## Abstract

**Background:**

Historically in Australia, all levels of government created collective wealth by owning and operating infrastructure, and managing natural assets, key public goods and essential services while being answerable to the public. This strong state tradition was challenged in the 1980s when privatisation became a widespread government approach globally. Privatisation involves displacing the public sector through modes of financing, ownership, management and product or service delivery. The Australian literature shows that negative effects from privatisation are not spread equitably, and the health and equity impacts appear to be under-researched. This narrative overview aims to address a gap in the literature by answering research questions on what evidence exists for positive and negative outcomes of privatisation; how well societal impacts are evaluated, and the implications for health and equity.

**Methods:**

Database and grey literature were searched by keywords, with inclusion criteria of items limited to Australia, published between 1990 and 2022, relating to any industry or government sector, including an evaluative aspect, or identifying positive or negative aspects from privatisation, contracting out, or outsourcing. Thematic analysis was aided by NVivo qualitative data software and guided by an a-priori coding frame.

**Results:**

No items explicitly reflected on the relationship between privatisation and health. Main themes identified were the public cost of privatisation, loss of government control and expertise, lack of accountability and transparency, constraints to accessing social determinants of health, and benefits accruing to the private sector.

**Discussion:**

Our results supported the view that privatisation is more than asset-stripping the public sector. It is a comprehensive strategy for restructuring public services in the interests of capital, with privatisation therefore both a political and commercial determinant of health. There is growing discussion on the need for re-nationalisation of certain public assets, including by the Victorian government.

**Conclusion:**

Privatisation of public services is likely to have had an adverse impact on population health and contributed to the increase in inequities. This review suggests that there is little evidence for the benefits of privatisation, with a need for greater attention to political and commercial determinants of health in policy formation and in research.

**Supplementary Information:**

The online version contains supplementary material available at 10.1186/s12992-024-01036-w.

## Background

Historically in Australia, all levels of government created collective wealth by owning and operating infrastructure, and managing natural assets, key public goods, and essential services while being answerable to the public. This was part of a strong state tradition [1] which was challenged in the 1980s when privatisation became a widespread government approach globally. In low and middle income countries this was imposed by the World Bank through Structural Adjustment Packages (SAPs) which have been shown to weaken the public sector and result in less accountable services [2]. In high income countries, privatisation was enacted by governments which adopted neo-liberal policy prescriptions; and by the 1990s Australian State and Federal Governments were privatising a significant portion of the public sector [3].

Privatisation involves displacing the public sector through modes of financing, ownership, management, and product or service delivery [4]. It encompasses outsourcing, whereby governments ‘contract out’ traditional public sector functions to private service providers. It also includes entering into public-private-partnerships (PPPs) for infrastructure projects which are financed and built by agreement with private corporations under long-term arrangements [5]. PPPs have a contested history in Australia, with a contract failure of over 50 per cent for hospital partnerships and do not lead to greater efficiency in service delivery [6]. There is a familiar ideological debate between promoters of publicly managed services and those favouring a stronger role for the private sector, with accountability arrangements for the private sector not clearly defined [7]. Democratic risks are identified as PPPs are long-term and may extend beyond a particular parliament [6].

Another mode is competitive tendering, or the process of selecting a preferred supplier from a range of potential contractors by seeking offers or tenders, and evaluating these against selection criteria [8]. Reports and academic literature on competitive tendering and contracting by public sector agencies in Australia have reviewed the benefits and costs, noting diverse claims about its effects [8, 9]. Competitive tendering expanded from basic services to core government activities such as human service provision including prison management, employment assistance, and hospital services [10]. Competition may improve quality in normal markets. However, there are costs involved with implementing reforms, and it has been argued that pro-competive policies are not a solution, and may even cause harm [11].

Governments and industry have continued to call for further privatisation and sale of public assets over decades, with a commitment to supporting greater private sector investment, streamlining tendering processes, reducing ‘red tape’, and promoting the sale of public assets towards other forms of reinvestment [10, 12–14]. Dissenting views have argued that the implementation of competitive tendering, including in the social welfare sector, results in a range of negative impacts including loss of autonomy, reduced collaboration, learning, choice and diversity, and increased administrative costs [9, 15].

Privatisation approaches now include contracting out high-level communication and information technology functions, and using public funding to contract major multinational consulting, legal and accounting firms to provide advice to governments on a wide range of policy issues including aspects of privatisation [16, 17]. Advocates argue that privatisation can act as a public good (eg.13), or that it improves accountability [18]. However, global changes including de-regulation, privatisation, and the entry of foreign capital has changed the relations between the state and the market [19].

The dominant view of privatisation is that it is largely as a government economic or fiscal technique, concerned with transferring activities and / or assets from the public to the private sector. Although correct, this is arguably a narrow and one-dimensional view, as it focuses exclusively on the financial sphere [20]. Whitfield [21] provides a typology to understand the ways in which public services and the welfare state are transformed by privatisation and marketisation across four domains. These relate to global public goods, privatisation of assets and services, privatisation of governance and democracy, and privatisation of the public domain. This includes the primacy of market values, and privatisation of public intellectual capital and public space [21].

Privatisation affects people’s access to the social determinants of health (SDoH), including secure employment, education, and transport [22, 23]. Furthermore, reduced government intervention in markets since the 1980s, from adoption of neoliberal policy approaches, has undermined oversight and control of the private sector’s influence on population health and equity [24]. Outsourcing of government functions to the private sector also has detrimental effects on the capacity of the Australian public service [25, 26]. The Australian literature suggests that when there are negative effects from privatisation, the harms are not spread equitably [27]. However, the health and equity impacts of privatisation appear to be under-researched, hence the need for a review of this literature.

## Methods

Our narrative literature review on privatisation of government services in Australia aims to address a gap in the literature by seeking to answer the following research questions:

- What evidence is there for negative or positive outcomes of privatisation?
- How well are the societal impacts of privatisation evaluated?
- What are the implications for health and for health equity from the privatisation of public services?

The review process was guided by Green et al., 2006 & Ferrari, 2015’s key features of narrative reviews [28, 29].

### Data collection

Data sources were the Web of Science, Proquest Central, Informit, and Scopus databases for peer reviewed literature, and Analysis and Policy Observatory (APO) and Google Scholar for grey literature. Search terms and the database search string were compiled by the co-authors and refined by a university subject librarian to capture the context of privatisation, outsourcing, and contracting out, together with the associated outcomes, results, impacts, consequences, effects, evaluation, successes, failures, or other measures:

Search string: ALL= (privati? ation OR outsourc* OR “contracting out”) AND ALL= (outcome* OR result* OR impact* OR consequence* OR effect* OR evaluat* OR success* OR fail* OR meas*).

Inclusion criteria were items limited to Australia, published in English between 1990 and 2022. Database searches captured some literature from the 1980s, but most was from 1990 onwards, and the search spanned 1990 to the start of the review in 2022. Inclusion criteria were any industry or government sector which included an evaluative aspect, or identified positive and/or negative aspects from privatisation, contracting out, or outsourcing. Documents were excluded if they did not meet the above criteria.

Database searches were augmented by selected grey literature in consultation with a university subject librarian. Australian Policy Observatory was searched on 26/7/2022 using the same search terms as for the databases, but limited to title only and for the period 2010–2022. Two hundred and seventy eight abstracts were screened and 16 items saved for full text reading. Google Scholar was searched on 2/8/2022 using the search term ‘Privatisation in Australia’. This resulted in 489 ‘hits’, with the first 350 reviewed before relevance declined. Seven items were saved for full text reading. The reference lists of several key articles were also checked for potential inclusion through snowballing. After screening, the items reserved for full text reading were saved in an EndNote library. A database search tracking sheet was compiled to record details of the data collection process.

Summaries of saved items were compiled to aid reflection on those most suitable for inclusion in the review [28] (See Table 1). Thirty six of the saved documents were selected on the basis that they included an evaluative aspect, or cited positive or negative aspects that may help to understand health and / or health equity impacts of privatisation. A brief overview of each item was recorded in a Summary Table as recommended by Younas & Ali [30]. (See Appendix 1). JA completed the selection process in consultation with co-authors in team meetings, and subsequent recommended selective literature searches augmented the background literature.

**Table 1:** Database tracking sheet

Search date	Database	Years searched	Number of abstracts screened	Items saved for full text reading
30/8/22	Web of Science	1990–2022	203	21
30/8/22	Proquest Central	1990–2022	157	25
30/8/22	Informit	1990–2022	204	42
31/8/22	Scopus	1990–2022	150	13

(Adapted from Green et al. 2006)

### Data analysis

All selected items were imported into NVivo qualitative data software to assist with thematic analysis [31]. An a-priori coding frame was developed by the co-authors and augmented by sub-codes reflecting the research questions (See Appendix 2). JA undertook the coding in collaboration with, and with verification by TF and FB in a team meeting. Insights from the coding were later discussed by all authors to help identify key themes.

## Results

The analysis revealed only five documents which mentioned (generally qualified) positive aspects of privatisation. No articles explicitly reflected on the relationship between privatisation and health. However, there were articles that described the impact of privatisations on known social determinants of health. Very few articles reported positive societal impacts and most provided evidence for negative effects on SDoH.

**Fig. 1:**
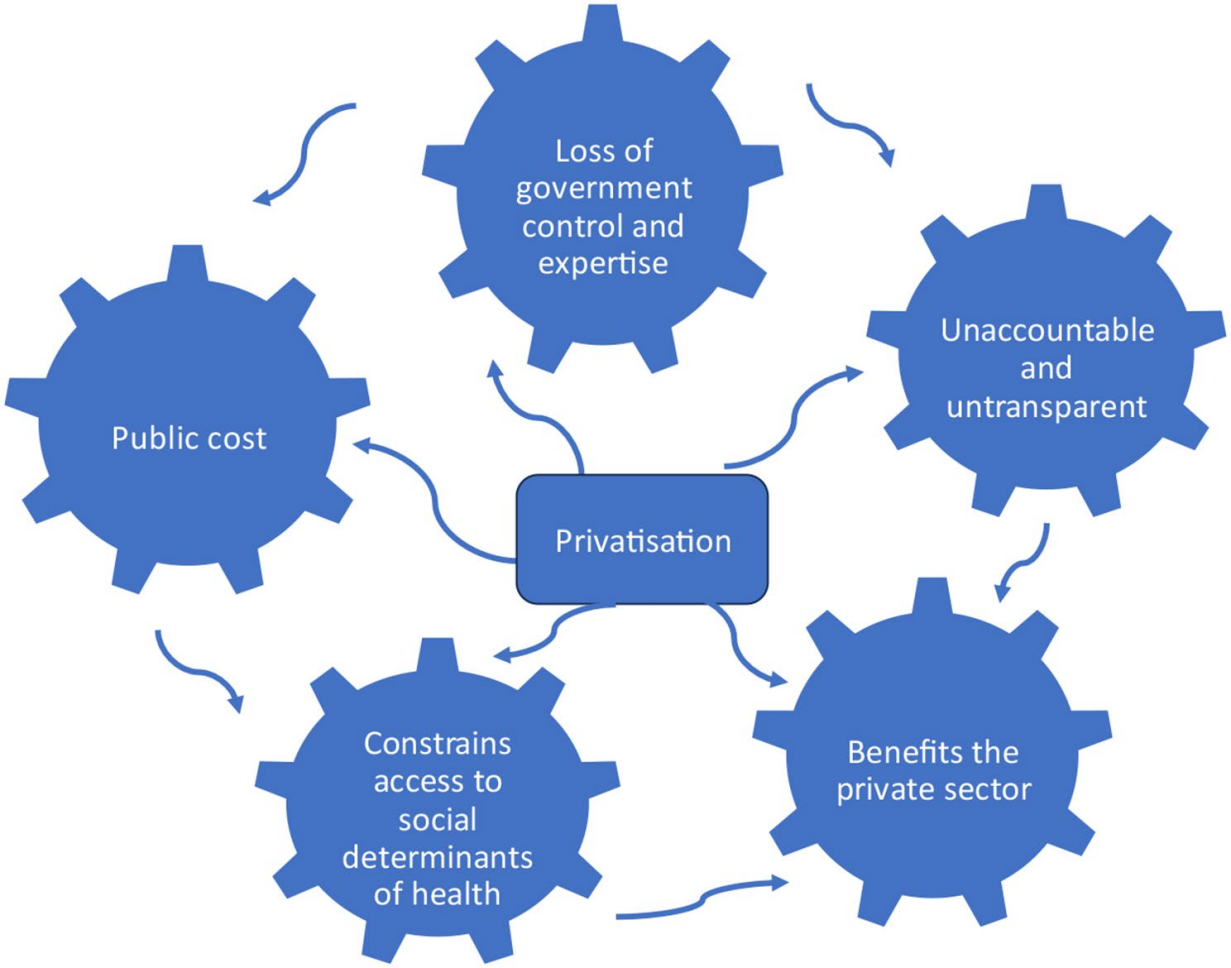
Privatisation key themes

The main themes identified were the (1) public cost of privatisation, (2) loss of government control and expertise, (3) lack of accountability and transparency, (4) constraints to accessing SDoH, and (5) benefits to the private sector (Fig. 1).

### The public cost of privatisation

One rationale for privatisation is the presumption of cost savings. However, in their survey of privatisation of Australian government enterprises since the 1980s, Abbott & Cohen [32] stated that few studies have been undertaken on the distribution of costs and benefits of restructuring and privatisation. Although privatisation can lead to increased efficiency, it may be difficult to distinguish between gains from increased competition, regulation, or privatisation. However where such gains are achieved, the costs and benefits are distributed unequally between consumers, employees and shareholders [32].

### Loss of government control and expertise

Loss of government control and expertise was highlighted in findings related to outsourcing health and social welfare provision. The impact of changes to service delivery models on the administration of Government programs was the focus of a 2020 Australian Senate Inquiry into the privatisation of state and territory assets and new infrastructure [33]. The committee heard that outsourcing human services has negative outcomes for the most disadvantaged Australians while also undermining the capacity of the public sector to design and deliver effective services [34]. Australia spends hundreds of million of dollars annually on private consultancies to deal with the impact of enforced public service staffing caps which in 2020, was equivalent to 12,346 public service roles [35]. With a reduced public service, COVID-19 management failings occurred in the rollout of vaccines, testing and tracing, and by the use of casualised and under-qualified workforce in aged care. As the policy report by Dyrenfurth [36] argues, this led directly to the COVID-19 crisis and deaths, especially amongst the elderly.

Tilley [37] explored the role of the automation and outsourcing of aspects of social security systems and its impact on recipients. In 2016, the Australian Government introduced a cashless debit card with the stated aim of reducing social exclusion and addressing social harms including gambling and substance abuse. It entered into a PPP arrangement with the private bank Indue Limited which was contracted to administer the automated payment system. During the first year of trialling, the card cost approximately $10,000 per participant to administer, with $128.8 million included in the forward estimates to expand the program [37]. As well as the financial impost, the relinquishing of primary control of the data to a private entity undermined the government’s exclusive powers of surveillance, with potential for further data-sharing. It changed the relationship between the citizen and the state and reproduced the social harms the card was purported to address [37].

### Lack of accountability and transparency

Lack of accountability and transparency were common themes in the review, including for privatised infrastructure projects, human service provision for community and corrective services, and for privatised employment and outsourced hospital services. In research into the privatisation of electricity and urban rail, and PPPs for road infrastructure in Victoria, Hodge & Coghill [38] noted negative impacts on democratic accountability to citizens and public institutions. The initial divestiture of electricity involved secrecy, and removal of prior rights to information under administrative law by weakening Freedom of Information laws [38]. Following the privatisation of urban rail, accountability to passengers was dramatically reduced.

Lack of accountability and transparency was also a key feature of human service provision revealed in their report into privatisation of prisons by Andrew, Baker et al. [39]. Australia has the highest rate of private incarceration per capita, with 20 per cent of the prison population held in privately-run facilities [40, 41]. Andrew, Baker et al. concluded that there was no evidence that private prisons are cost effective. There is a lack of state uniformity, evidence of improved performance and efficiency gains are incomplete and lacking transparency, there is poor public reporting, and the true cost of private incarceration remains unknown [39]. While accountability systems and performance management have become more sophisticated, publicly available information allows for little real scrutiny.

Other researchers also note that commercial-in-confidence provisions ‘cast a shroud of secrecy’ over Victoria’s privately operated prisons, undermining the ability to identify contractual violations or potential remedial actions (42 p. 228). A major constraint to accountability is the lack of an independent body responsible for oversight, with Victoria maintaining an ‘in-house’ review and monitoring scheme which lacks public transparency [42].

In their report on the impact of privatisation and outsourcing on community services, Mitchell et al. [43] explain that although outsourcing has been applied to a wide range of public services, public reporting is rare. PPPs involve complex contracts, but the costs to the state are obscured by ‘commercial in confidence’ declarations.

Lack of transparency and accountability were also highlighted in the provision of privatised employment services, with Rogers [44] reporting that it may be difficult to determine to whom not-for-profit organisations are ultimately accountable. Although outsourced employment contracts define obligations to government, organisations may also have obligations to service users, religious entities, or financial backers [44]. Tensions therefore arise between not-for-profit service providers’ values and government employment policies [44].

Young used empirical evidence and theoretical literature to discuss outsourcing of several support services for a large Victorian hospital in 1997. This study found a lack of transparency and minimal financial reporting, with service quality sacrificed to reduce costs. Management by contractual arrangements was deemed problematic [45]. The McKell Institute also investigated the ownership structure of the health system and identified that privatising public assets is a business ‘fraught with risk’, especially in relation to healthcare. Critically, it was argued that a broad consideration of the merits of widespread health privatisation demands that policy makers first determine whether further privatisation risks eroding the concept of universal healthcare and whether it could threaten equity of access [46].

### Constraints to accessing social determinants of health

Social factors including employment status, education and income level strongly influence a person’s health [47]. There are wide disparities in the health status of different social groups in all jurisdictions [47], with social and economic inequities leading to health inequities [48]. The review found wide-ranging constraints to accessing the social determinants of health spanning privatised employment services, education, human rights, and infrastructure.

#### Employment services: job seekers

Employment is a key SDoH [49], with the review highlighting a range of constraints for both unemployed people and service providers. The Commonwealth Employment Service was privatised in 1998 under the Howard Government. Within the outsourced employment services regime which followed, unemployed people aged 18 to 29 years, registered with Job Services Australia (Workforce Australia), were required to undertake a work experience program called ‘Work for the Dole’ [50]. The rationale was to provide job seekers with services needed to acquire new skills and to improve their chances of finding paid employment under Australia’s ‘mutual obligations’ policy framework [51]. In their evaluation of the Work for the Dole scheme between 2014–2015, Kellard et al. [52] noted certain positive and negative aspects of this active labour market program. However, the term ‘work for the dole’ reflected legacy issues that associated the program with undertaking menial tasks such as graffiti removal, and was stigmatising and unhelpful overall for engaging job seekers. Work for the Dole was perceived to be punitive, and a source of free labour, rather than for prioritising provision of relevant work experience for job seekers [52].

A competitive tendering framework encourages service providers to meet the specified goals in the most cost-effective manner [53], but with perverse incentives. These result in activities referred to as ‘creaming’ or ‘cream skimming’ (focusing on more able clients with better employment prospects, and ‘parking’ (under-serving harder to place clients). These are forms of ‘adverse selection’ where clients are chosen for assistance in inverse proportion to need [53, 54]. Service providers are given incentives to seek out those clients whose needs can be more easily met, with other job seekers diverted to ‘providers of last resort’, or receive no service at all [53]. These findings accord with the view that privatised businesses are less sensitive to the situation of poorer ‘customers’ who are not directly profitable to serve [55].

Research by Moore [56] found that young people are another disadvantaged job seeker group who are served inequitably under privatised employment services. They experience vocational barriers including limited opportunities for work experience, difficulties accessing transport, and high incidence of mental health problems and family issues [56]. However, evaluations of Australian employment services have often neglected to consider young job seekers, despite a persistently high youth unemployment rate. Moore [56] also notes that three evaluations of the Job Active program found that although there were some cost savings, not all young job seekers were benefitting equitably. However as also noted, it was people living with disabilities and Indigenous job seekers who were the most negatively affected cohorts.

Other research on employment services by Burgess showed that market-style operations are unhelpful for unemployed people who lack the necessary resources to find a job [57]. Real choice for service users was limited by a lack of information about service providers, and decisions could be over-ridden by Centrelink, the government agency which is responsible for service allocation. As Burgess notes, with publicly-funded provision there is no real market, but a contrived or quasi-market. The participants are the Federal government as the ultimate purchaser of services, agencies which sell services, Centrelink as allocator of services, and Job seekers who are the service users. Within the privatised regime there is thus an inherent tension between price and quality of services [57].

#### Employment services: employees

Employees are also affected by privatisation, with some service providers facing internalised conflicts over their roles. For over a century in Australia, faith-based organisations have traditionally provided welfare services to disadvantaged populations. However, interviews with faith-based service providers in outsourced agencies [58] revealed the challenge of being unable to fulfil their distinctive holistic core missions and express their values when governments prioritise market-based approaches to contracting out. The challenge for these agencies is deciding whether to accept ‘tied’ government funding, and if they do so, find ways to adapt to protect their values, seek alternative funding sources, or withdraw completely from service delivery [58]. This potentially limits options for citizens in vulnerable circumstances.

Mitchell et al. [43] explain that while transnational corporations (TNCs), or for-profit providers, play a large role in delivering outsourced services in Australia, the not-for-profit (NFP) sector, which operates from a different financial and values base, has also embraced government services provision, but in doing so, lose their capacity to advocate to government on behalf of their traditional clientele. Rogers [44] discusses the need for staff in NFP service provision to increasingly focus on financial aspects of the organisation, whereby workers may become less responsive to job seekers and more rule-bound.

Some NFP service providers claim that they are conflicted by the need to impose demerits and financial penalties for non-compliance by disadvantaged and vulnerable people. Some organisations also choose not to challenge aspects of government policy for fear that their employment network may be negatively affected [44]. Staff report unhappiness also with the high administrative load and general systems operations. The NFP sector relies heavily on volunteers but there is limited information on any implications for workers, volunteers and organisations [44].

The review of the literature highlighted employment constraints in other human service sectors and employment cohorts, including employees. In their research on austerity, staffing inadequacy, and contracting-out in aged care, Farr-Wharton et al. [59] found that employees who work in a facility that has inadequate staffing and offers low peer-support often seek alternative employment. This leads to increased workers compensation claims and retention costs that are externalised to the taxpayer.

Negative outcomes for employment also occur under the correctional services regime. This has resulted in poor outcomes for prison workers in most states from staff cuts as part of the privatisation process [39]. In Western Australia, the Economic Regulation Authority (ERA) argued that a benefit of introducing private providers was a reduction in the costs of workers’ entitlements, with prisoners viewed as ‘stakeholders’, but prison officers as ‘a cost to that system’ (39 p. 5).

#### Education

Education providers identified in the review include Technical and Further Education (TAFE) and early childhood education and care (ECEC). Rodd researched the experiences of TAFE workers from the transformation of the vocational and training sector (VET) between 2012 and 2017 [60]. Under these changes students became ‘customers’, and VET service providers were forced to compete for market share. The research revealed significant economic cost for taxpayers, with students left heavily indebted and bearing the major cost of privatisation [60].

Once largely managed by the community sector, ECEC services have also shifted to for-profit providers. This has driven down operational standards, reduced access to care, and imposed a cost to taxpayers [61]. The Australian Council of Trade Unions (ACTU) notes that these workers are often early victims of cost-cutting and profit seeking which leads to underpayment, overwork, and insufficient support.

#### Unions

Unions are arguably an unappreciated SDoH [62]. They help to raise wages, decrease inequality, decrease discrimination, improve workplace safety and affect other health determinants [62]. The review identified research revealing the impact of privatisation on union membership. Oliver [63] examined two Western Australian unions and found that privatisation was a key factor in declining union membership and union power. A ‘clearing out’ of ageing workers occurred in some industries under both major political parties in preparation for privatisation, leading to large job losses and a loss of union culture [63]. This specific case supports the more general correlation between unions and access to SDoH in the form of higher wages, lower income inequality and other factors [62]. Research by Young found that while privatisation resulted in some cost savings it also led to a reduction in union power, the nature of the relationship between contract and internal staff, and service quality [45].

#### Human rights

Human rights are key to addressing inequities in SDoH [64]. The review identified constraints to human rights due to the privatisation of Australian immigration detention facilities, and from workers’ rights to compensation. The management of Australian immigration detention facilities was outsourced from 1998 under a range of successive contractual regimes. In researching the opportunities and challenges for implementing human rights within Australian privatised detention centres, Penovic [65] notes that even though the Federal Government cannot outsource its common law duties or international human rights obligations, the removal of direct ministerial responsibility can obscure government responsibility for human rights abuses when it is distanced from its own policies. Australia’s detention regime is deemed to be abusive and inconsistent with human rights, and Penovic notes these features are exacerbated by the privatisation of management [65].

Crowley-Cyr [66] notes that although the state’s duty of care for those under its control and supervision cannot be delegated, contemporary contractualism legitimates social exclusion by focusing only on the purchase of outputs, rather than the delivery of outcomes. This focus limits the extent of an individual’s contract with the state and has led to violations of immigration detention standards and operating procedures of a contractor (GSL). This was in respect of receiving appropriate medical assessments and treatment for injuries, being denied basic amenities, and that detainees were ‘humiliated and treated in an inhumane, unsafe and undignified manner’, and with the application of undue force during transportation (67p. 95).

In 1999, the Western Australian Parliament passed the Court Security and Custodial Services Act which allowed for outsourcing prisoner transportation, with prison management transferred in 2007 to GSL Custodial Services Pty Ltd (GSL) (now known as G4S) which provides privatised services to the Australian justice system including correctional facilities, courts, police custody, electronic monitoring, prisoner transport and offender rehabilitation [67] A Coroner’s Inquiry followed the death of an Indigenous Elder by heat exhaustion in 2008 during a prison transfer by G4S. The coronial findings noted a lack of policies and procedures, inexperienced staff, and a lack of oversight from the Western Australian Department of Corrective Services [68].

The 1966 human rights Covenants, the right to work and rights in work, are addressed in the International Covenant on Economic, Social and Cultural Rights [69]. Workers’ compensation in event of workplace injury, disease or death is a critical determinant of health for workers and their families, with many employers acknowledging financial wellbeing as a SDoH for workers [70]. A case study of outsourcing claims administration for the South Australian workers’ compensation scheme, conducted by Purse [71], found that outsourcing failed to meet its objectives, that employees’ interests and rights were often subordinated to those of employers, and that the system lacked accountability.

#### Infrastructure

Wide-ranging privatisation of infrastructure has occurred in Australia with this review noting telecommunications and banking services, ports and airports. Privatised markets require regulations to protect people’s interests. Despite the ideological similarity of the goals of privatisation and de-regulation, Stretton [72] noted that privatisation has generally led to increased regulation. Webster [73] conducted research into the performance of regulation in the Australian Telecommunications industry during a period of privatisation (six months between 1999 and 2000), finding that rural customers were disadvantaged in receiving services. The regulatory focus was on performance; doing mainly what is measured in order to meet compliance, while neglecting other important areas of customer service [73]. Regulations thereby failed to ensure a universal standard of service.

The National Partnerships Agreement on asset recycling initiative (ARI) provides incentives to State and Territory Governments to sell government-owned assets to reinvest in economic infrastructure [74]. In their 2014 submission to a Senate Standing Committee on the privatisation of state and territory assets and new infrastructure, the Australia Institute [75] argued that the ARI is built on a presumption, without empirical basis, that privatisation is always preferable, and highlighted that many of its advocates have vested interests. They note that since the Commonwealth Bank was privatised, many retail bank branches in regional areas have closed because they did not deliver the very high rates of returns required of the privatised bank, and this has led to poor service outcomes [75].

Privatisation of infrastructure including Australian ports has also adversely affected local employment [76]. Prior to privatisation, port users including union members were board directors. However, the boards are now skewed towards business representatives such as from investment funds [76]. Although private port companies often offer a guarantee of no job cuts within a specified timeframe, evidence shows a reduction in the workforce at privatised ports once this time elapsed. Research shows a 31 per cent decrease in the number of employees at the Port of Brisbane due to job losses from workers who left the operations not being replaced, and through contracting out of maintenance work [76].

Job losses were also identified in research by O’Donnell et al. [77] on the privatisation of Sydney Airport. The Federal Labor Government led the privatisation process for federal airports, negotiating with five unions in 1995 so that successful bidders maintained existing airport staff and their wages, employment conditions, and entitlements for a period of 12 months. However, one year after the sale, 40 per cent of the workforce (160 employees) were made redundant in order to reduce costs [77] This underscores the negative impacts on unions reported earlier in this review.

### Benefits to the private sector

A key theme emerging from the review was the many ways by which privatisation benefits the private over the public sector. These benefits accrue from hospital and wide-ranging infrastructure privatisation and the scope of outsourcing services to large transnational corporations (TNCs). These entities also benefit from engaging in tax minimisation strategies in undertaking their roles. As uncovered in their report on hospital privatisation, the McKell Institute showed that private operators may choose to only manage the most profitable services, leaving the public sector to undertake the more difficult and costly work. The ultimate responsibility for failures associated with social infrastructure projects is borne by governments when a private partner is either unable or chooses not to uphold its contractual obligations [46].

Case studies of contracting out undertaken by Quiggin [53] covered the scope of road contracting, school cleaning services, water and employment services, Commonwealth information technology services, and the Commonwealth Serum Laboratories (CSL). Quiggin found that presumed public benefits of contracting out have been overestimated. There may be benefits in cases where peripheral government risks can be successfully transferred to a contractor who is able to manage those risks, but badly designed contracts can result in governments, and hence the community, bearing high risks while gaining no return. Such policies may therefore reduce rather than enhance public welfare and so impact adversely on health and health equity [53]. Quiggin argued for consideration of a return to public ownership in some instances [78].

The privatisation of electricity networks also benefits the private sector due to the operations of ‘gentailers’. These are companies which are both retailers and generators which allows them to take advantage of both wholesale and retail markets, with governments assuming responsibility for maintaining costly and unprofitable aspects of operations, including poles and wires [79]. The benefit of privatisation to the private sector from the privatisation of electricity is also noted in research by Cahill and Beder [80]. Large corporate electricity consumers in Australia received the greatest gains during the 1990s, having successfully lobbied government for guaranteed fixed prices which were effectively subsidies by the broader population.

Some large corporations also gain significant financial benefit from being contracted to provide multiple public sector roles. In a commentary about the growing power and influence of one large TNC, O’Keefe [81] notes that Serco, [revenue £4.425 billion 2021] is contracted by governments for roles in hospitals, prisons and prisoner transport, immigration detention centres, military logistics, military health support, traffic management, health, justice, and other arenas. The only common theme across this wide portfolio is that these roles were all previously undertaken by governments [81].

Privatisation has also created benefits for investment banks which can impose multiple charges for the one transaction. This includes setting up, then managing funds and individual assets [79]. Other financial benefits accrue to the private sector when companies that adopt aggressive tax avoidance strategies are still rewarded with lucrative government contracts [33]. This has implications for population health and equity due to the loss of government revenue for health and social investment.

In their article on the privatisation of custodial services, Baldino et al. [68] highlight that training requirements for the private security industry remain limited and inconsistent, and include a strategy of ‘lowballing’. This allows private companies to benefit by using low bids to gain a government contract which is then re-negotiated at a higher rate [68]. Such practices mask the benefits of privatisation to the private sector and counter claims of cost-effectiveness for the public sector.

Collyer et al. who undertook nine case studies of privatisation during the 1990s concluded that those who gained most from privatisation were those with resources and influence, while those who were most negatively affected had no opportunity to even engage in the decision-making process [27]. These researchers identified that clear winners included politicians and political advisers; consultants and associated businesses; banks and financial institutions; high-income employees; political parties; investors and new owners; consumers and customers. Clear losers were low-income and non-management employees; citizens and the state [27].

## Discussion

**Fig. 2:**
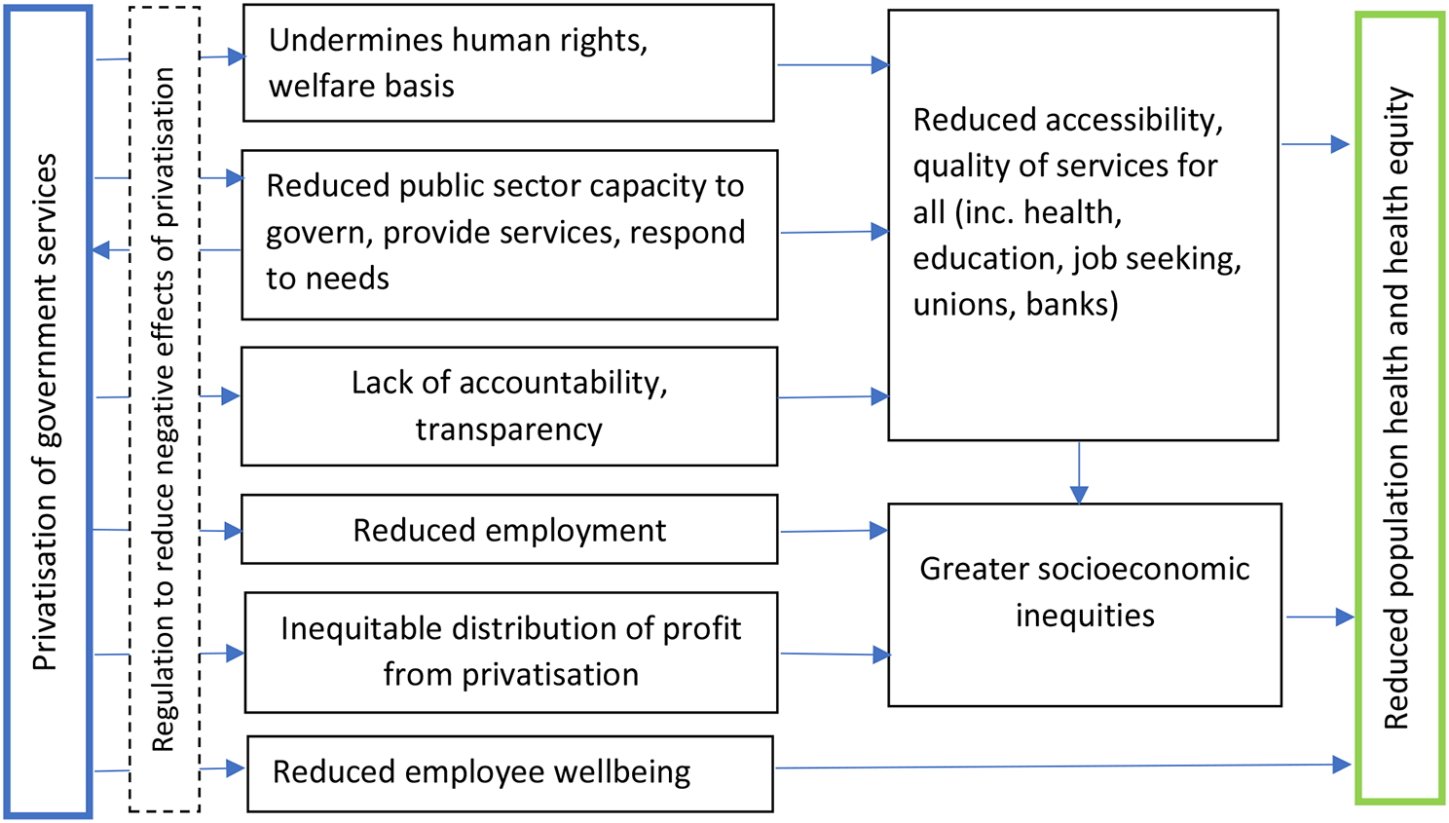
Pathways from privatisation to health and equity

The main aim of our narrative review was to identify the impacts privatisation has had on health. As Fig. 2 depicts, these pathways include the undermining of human rights, the need for increased regulation, the long term implications of a reduced public sector capacity, the shift of public sector funds to private profits and reduced employee wellbeing.

Since the 1980s, Australia and its economy has been radically transformed by the processes of privatisation, de-regulation and marketisation, with an ideological adherence to the hollowing out of the state [16]. In Australia, the latest available statistics reveal that privatisation saw government-owned enterprises dropping from 7% of gross domestic product (GDP) in 1989-90 to 1.3% in 2011-2 [82]. They reveal that between 1987 and 2012 the proceeds of privatisation in Australia amounted to AU $194 billion dollars [83]. This narrative review of privatisation describes the running down of public services and the advantages given to the private sector actors who take on the divested roles. Whitfield argued that ‘privatisation is more than asset stripping the public sector; it is a comprehensive strategy for permanently restructuring the welfare state and public services in the interests of capital’ (1983: 1–2).

Privatisation and marketisation, or the introduction of competition into public services, are interlinked, with the latter creating the overarching social and economic conditions that foster further privatisations [21]. Our review suggests that we found evidence for each part of Whitfield’s four part typology [21]. For example, the marketisation of public goods includes public health, while the privatisation of assets and services includes private finance of infrastructure and services under PPPs. Privatisation of governance and democracy includes the transfer of services to arms length companies and the corporatisation of quasi-public bodies. The public domain encompasses the replacement of public service values and principles by market ideology and commercial values [21]. This implies that the impacts of privatisation in Australia has been more than the sum of its parts because the broader aim of undermining the public sector has been achieved.

Privatisation is both a political and commercial determinant of health: the systematic process of distributing resources, structuring relationships, administering power simultaneously in ways that ‘mutually reinforce or influence one another to shape opportunities that either advance health equity, or exacerbate health inequities’ [84p. 9, 85]. As the main responsibilities of the private sector are to their shareholders, the private sector should not be presumed to act in the public interest. Governments are primarily responsible for ensuring that the public interest is accounted for when determining any form of privatisation [86]. As this is not always accepted by governments, there have been cases where there is a lack of public compensation from divestments, due to weak regulations [86]. Governments have a responsibility to regulate privatisations and also to evaluate whether the intended benefits are actually achieved. The finding that ministers generally maintain an ‘arms length’ approach to privatisation shows the lack of political will to regulate for health and equity outcomes [38, 65].

The case studies by Hodge and Coghill [38] found that while privatisation per se may not have cost votes, prioritising of managerialist values over public accountability did. This highlighted the paradox, that when privatising government operations it is necessary to strengthen mechanisms of public accountability. This is particularly the case if a government wants to follow an equity agenda. Equity outcomes are generally achieved when public policy takes proportionate actions in favour of groups that are disadvantaged in universal systems [49, 87]. Our review found no evidence of equity considerations being built into privatisation projects.

As the research by Collyer et al. [27] discovered, those who benefit from privatisation are mainly those with existing resources and influence. Those for whom privatisation has negative impacts are those who are unable to participate in decision-making even though they have the most to lose. Costs and benefits of privatisation have therefore been borne inequitably, with a clear transfer of public funds and resources to the private sector with health inequities flowing from these social and economic inequities [48].

Given that different forms of privatisation and impacts are so embedded in the current political landscape, these are difficult to overcome. In the short term this will require fairer contracts, better governance, accountability, and regulation. In the longer term this may require assets and services being brought back into government control. Remunicipalisation, de-privatisation’ or ‘in-sourcing’ are terms which refer to the process of bringing privately owned and/or managed services back into full local government ownership, management, and control [88]. The term municipalisation refers to the establishment of new public services and institutions to meet collective needs [89].

Arguably, in 30 years of privatisation in Australia, there is no instance of privatisation where the public would have benefitted as much as if the asset remained in public hands [90]. Instances of privatisation failure deny the notion that privatisation is irreversible, with the call for a rational and systematic re-evaluation of the appropriate roles of the private and public sectors [78]. While political actors often view privatised infrastructure as an unchallengeable necessity, the general public has largely voted against privatisation whenever the opportunity arises [91].

As Spoehr contends, the experience of COVID-19 has particularly challenged the privatisation narrative, forcing government to re-evaluate the role of the public sector; with the marshalling of public services, including the public health system, community services, support systems for income and business, and the police [92]. A strong case now exists for renationalising a broad scope of public assets including electricity transmission, airports and roads [90]. For example, recently announced was the intention to revive the Victorian State Electricity Commission to fast-track de-carbonisation as the government lacks confidence that the private sector can do so in a timely manner [93].

Reversing privatisation is not easy. However, there are also other ways to bring back private services into public control. One is to abolish compulsive competitive tendering to eliminate the role of bidding by the private sector [94]. Another is policy reform to prioritise public ownership and control over privatisation [95]. The need to restore public staffing levels highlights the importance of bolstering state capacity [96].

## Conclusion

This review suggests that there is little evidence for the benefits of privatisation, with a need for greater attention to political and commercial determinants of health in policy formation and in research. We can conclude from our review that evidence exists that privatisation is likely to have had an adverse impact on population health and contributed to the increase in inequities over the period of privatisation, and so of health equity.

### Electronic supplementary material

Below is the link to the electronic supplementary material.

Supplementary Material 1

Supplementary Material 2

## Data Availability

All data is included in the paper.
