# Supplementary material for: Privatisation of government services in Australia: what is known about health and equity impacts
Source: Global Health. 2024 Apr 16;20:32. doi: 10.1186/s12992-024-01036-w (PMC11020887; doi:10.1186/s12992-024-01036-w)
Supplement: Supplementary file 1 — Supplementary Material 1 [file 12992_2024_1036_MOESM1_ESM.docx]

**Appendix 1: Overview of reviewed sources**

| **Authors** | **Purpose** | **Type of source** | **Summary** |
| --- | --- | --- | --- |
| Abbott & Cohen, 2014 | To evaluate the compiled evidence on the success or failure of privatisation for improving efficiency across various industry sectors. | Survey research | Privatisation can lead to increases in efficiency, and increasing competition drove efficiency gains in some cases. However, in others it is difficult to distinguish between the gains from privatisation, regulatory reform, and increased competition. Where gains were achieved (from either privatisation or increased competition), generally the costs and benefits were shared disproportionately between employees, consumers, and shareholders. |
| Andrew, Baker & Roberts, 2016 | To describe the evolution of Australian private prisons. | Report | Overall, there is insufficient evidence to support claims favouring prison privatisation in Australia. Therefore, no further privatisations should take place before appropriate information is made available to policy makers and the public. There is need to properly assess the impact of privatisation and for more research that engages directly with those who are impacted: prison employees and prisoners. |
| Aulich, Jones & Head, 2016 | To explore how the public mission of public enterprises has transformed in recent decades from a role as nation builder to one as enabler of services. | Qualitative research | Based on the few available studies of post-privatisation performance of different entities, there is little evidence to suggest that change of ownership alone has yielded superior performance. This underlines long held views that differences in performance between public and private organisations seem to depend less on ownership and more on other factors such as size, task, regulatory environment, management skill and technology. |
| Australian Council of Trade Unions, 2016 | To provide several Australian case studies of publicly funded sectors where privatisation has been applied, either in whole or in part, and describe the achieved outcomes. | Submission to the People’s Inquiry into Privatisation | The entire privatisation agenda needs to be re-examined based on the evidence from almost 40 years of practice. While there are cases where positive outcomes can be achieved, this is by no means guaranteed. Where governments intend to pursue a privatisation agenda, there should be a process to ensure that privatisation, or contracting of government services, delivers positive outcomes for workers, service users and the government. |
| Baldino, Drum & Wyatt, 2010 | To consider the role of accountability mechanisms for improving contracting out of correctional and custodial services, and whether existing oversight frameworks can provide proper supervision and quality control of private security operators. | Qualitative research | Ultimately the circumstances of a reported death of a prisoner in contracted out transit services represented a failure in implementation of appropriate policies and processes. While the decision to contract out was not inherently flawed, private sector contracts do not absolve governments from duties to their citizens. |
| Bennett, Dawson, Lewis, O’Halloran & Smith, 2018 | To critically examine the current (privatised) mainstream employment services system, (Jobactive), and assess the experience of unemployed workers within the system against its stated objectives. | Report including case studies | Privatised employment services in Australia are unfit for purpose. They fail in the primary task of assisting people to find secure, long-term work. The structure of the contracted service between government and private providers is skewed towards providing job services staff with incentives to prioritise compliance measures over genuine assistance to people to move quickly from unemployment benefits into paid work. |
| Burgess, 2003 | To review the model underpinning the Job Network privatised employment services | Theoretical paper | The one-size fits all approach to unemployed people neither meets the diversity of needs of job seekers nor accounts for local conditions. Prescriptive practices may reduce flexibility and choice, while there is also the need for access to a minimum range of services. |
| Cahill & Beder, 2005 | To examine the realities of neo-liberalism in practice through an analysis of the history and experience of electricity privatisation in Australia. | Theoretical paper | Electricity privatisation and deregulation in Australia has been encouraged, facilitated, and implemented by governments and state agencies. The associated costs have been borne by the electricity sector workforce, which has been decimated, and by the rural and residential consumers, who have experienced resulting electricity prices rises. |
| Chen, Pateman & Sakalayan, 2017 | To present the latest trend in Australian port privatisation, long-term leasehold sale, and discuss its impact on port governance structure and port management. | Case study analysis and content  analysis of secondary data. | Concerns are raised by relevant port stakeholders about the long-term effects of privatisation. These include the risk of undervaluing port assets, increased charges, impeded competition, decreased long term port investment, and other issues affecting public interests. |
| Collyer, Wettenhall & McMaster, 2003 | To provide an overview of findings from the Australian Privatisation Study funded through the Australian Research Council | Article reporting research findings. | Privatisation does not produce better financial outcomes for the state and its citizens. The impact of privatisation is shown to maintain and exacerbate existing inequalities. |
| Crowley-Cyr, 2005 | To examine contractualism and exclusion in Australian immigration detention centres | Theoretical paper | Contemporary contractualism is an ideology that legitimates exclusion by limiting its process to the purchase of outputs rather than the delivery of outcomes. It allows the state to be insulated from the political and economic consequences of its coercive practices with negative implications for detainees. |
| Dyrenfurth, 2021 | To probe and compare previous instances of privatisation and contracting out over the last 30 years. | Policy report including case studies | Australia has a mixed experience with privatisation. The report questions and critiques governmental assumptions that privatisation and contracting out always leads to better outcomes. |
| Farr-Wharton, Brunetto, & Xerri, (2020) | To examine austerity, staff inadequacy, and contracting-out social services in aged care | Study using multi-level statistical analysis | This study provides insight into policy implementation in  relation to contracting-out, showing the cascading impact of contractors’ decision-making on employees’ outcomes, when the original policy is under-funded. It provides new insight into the limitations in contracting-out. |
| Hewson, 2021 | To argue that a major weakness of most privatisations has been the failure of governments to specify up-front and in adequate detail the service to be provided by the private owner or operator. | Newspaper article | There has been undue focus on maximising revenue from the sale of assets and businesses. There has also been poor implementation that has ignored the issue of competition, mostly to the detriment of the consumers whom privatisation was supposed to benefit by the provision of more cost-effective and accessible services. |
| Gallet, 2016 | To examine the challenges confronting Christian-based  organisations operating in the employment services quasi-market in Australia. | Qualitative research using semi-structured interviews | This paper examines the findings from a study of four Christian-based organisations contracted to deliver privatised employment services, specifically focusing on the influence of marketisation on the mission, culture and traditional values normally attributed to these organisations. Central to this investigation is the extent to which mission drift has occurred and church groups must decide whether, or not, to accept government funding to help them to fulfil their mission, seek alternative sources of funding, find new ways of adapting to protect their values, or withdraw from delivering services which they believe to be part of their core mission. |
| Hodge & Coghill, 2007 | To consider notions of accountability and the degree to which privatising public sector activities might either be improved or undermined. | Case study research | Accountability in the privatised state is characterised by lower political accountability but higher managerial and market accountability. The degree to which consumers or other constituent groups have seen accountability improvements is case dependent and not generalisable. While privatization activities per se may not have been a political vote loser, the elevation of managerialist values above the values of public accountability was. |
| Kellard, Honey & McNamara, 2015 | To undertake an evaluation of the Work for the Dole 2014-15 Programme. | Evaluation commissioned by The Dept of Employment using qualitative and quantitative research. | The title ‘Work for the Dole’ was generally agreed to not be helpful in engaging job seekers (and to an extent, new host organisations). There is clearly stigma associated with the programme and an assumption by some job seekers that Work for the Dole is punitive. It is seen as ‘working for benefit’ or providing ‘free labour’ rather than as opportunity to gain work experience, skills, and a reference for future employers. |
| Mitchell, Cook & Quirk, n.d. | To investigate the impact of State Government fiscal policy and privatisation on the quality of state public services, and the impact these policies have had on the number and quality of public services in States. | Selective summary of report commissioned by the CPSU/CSA and the other state public sector unions in Australia. | After 35 years of public sector retrenchment there is little evidence to support the claim that outsourcing and privatisation improves the quality and lowers the cost of providing what were useful public services.  The justification for cuts to useful public services has no evidential basis. |
| Moore, 2019 | To investigate the Jobactive privatised employment scheme and young job seekers and consider strengths, limitations, and suggested improvements to current practices | Qualitive research using interviews | Although high competition for entry level and low skilled jobs impacts significantly on a young person’s transition from education to employment, little research has explored the efficacy of the employment services provided to young job seekers to assist them to become more competitive in the labor market and to achieve sustainable employment. The current study has addressed this gap by exploring the strengths and limitations of the Jobactive program from the perspectives of young job seekers and employment officers. |
| O’Neill, Sands & Hodge, 2020 | To explain how public–private partnerships were used to create a mixed public–private prison system. | Case study research | Research conclusions are limited concerning costs, performance, and accountability due to data limitations. Based on available data, four proxies (assaults in prisons, hours out-of-prison cells, vocational education and training, and recidivism) did not indicate improved performance since the advent of Public Private Partnerships (PPPs). However, data for unnatural deaths, employment in prison, and prison escapes suggested real improvements. |
| O’Donnell, Glennie, O’Keefe, & Kwon, 2011 | To examine the consequences for consumers, airport employees, and the major airlines of privatising Sydney Airport. | Theoretical paper | The privatisation of Sydney Airport has impacted on employment conditions and airport employees’ job security. Following a 12-month period post-privatisation, where employment conditions were guaranteed, Sydney Airport management commenced a process of job cuts and the contracting-out of key airport functions, including security, baggage handling and maintenance. The aftermath of privatisation has resulted in labour shedding, outsourcing and a focus on cost minimisation by the airport's management. |
| O’Keefe, 2011 | To document the power and influence of the global Serco corporation which manages immigration detention centres and many other outsourced services in Australia. | Commentary article | Serco is mainly employed to undertake general government work. However, it has become very involved in government operations to the extent that lines between contractor and decisionmaker have become blurred. This is against the public interest. |
| Oliver, 2014 | To examine whether privatisation has contributed significantly to falling trade union density and membership in Western Australia. | Qualitative research | Privatisation in the two Western Australian unions was preceded by massive job losses and a resulting decline in union membership and density, but also a loss of union culture. |
| Penovic, 2014 | To examine the impact on human rights from outsourcing management of prisons and immigration detention centres. | Qualitative research | Concerns associated with privatisation relate primarily to cost cutting associated with the profit imperative, the removal of direct ministerial responsibility, and insufficient transparency and monitoring. |
| Public Services International, 2018 | The purpose of this inquiry is to begin a conversation about the issue of privatisation: including outsourcing, social impact bonds, user-pays, vouchers, commissioning, etc. and to build consensus around an alternative vision for our public services. | Report | Discusses the multifaceted consequences for privatisation on a range of sectors, including aged care, child care, hospitals, child protection services, VET, disability service, and prisons. |
| Purse, 2009 | To use the South Australian outsourced workers’ compensation scheme as a case study to address whether purported benefits of outsourcing are soundly based. | Qualitative research | The outsourcing of claims administration functions by the South Australian workers’ compensation scheme has been a costly failure. This is due in part to administration costs increasing significantly, no improvement in employer choice, with service delivery to injured workers in key areas compromised |
| Quiggin, 2002 | To argue that the benefits of competitive tendering and  contracting have been overestimated, and that many apparent benefits reflect transfers rather than efficiency gains. | Case study research | Appropriate use of competitive tendering and contracting can improve the efficiency of public service provision and the allocation of risk. However, the recent popularity of contracting out as a policy has resulted in numerous instances of inappropriate, poorly designed, and poorly implemented contracting out. The benefits of budgetary cost savings have been outweighed by losses in wages, reductions in working conditions, and reduced quality of service in some instances. |
| Quiggin, 2002 | To consider the issues of privatisation and re-nationalisation | Book chapter | Criticisms of privatisation have been the subject of strong debate, but until recently, arguments that the appropriate response to the failures of privatisation is a return to public ownership have simply been ignored. This position is slowly changing, although the public debate is behind the events in global jurisdictions. |
| Richardson, 2015 | The purpose of the submission is to comment on an inquiry into the privatisation of state and territory assets and new infrastructure or the “Asset Recycling Program”. | Submission | Privatisation is not always beneficial and can lead to poor outcomes for stakeholders and the wider public interest. The government should not be offering a blanket incentive for privatisation which will encourage sales of important assets for the public. |
| Rodd, 2021 | To examine neoliberal reforms of the vocational education and training (VET) sector in Australia, using Victoria as a case study. | Qualitative research using interview data | This case study confirmed previous theoretical arguments that neoliberal reforms in education have had a significant economic cost, wastage of taxpayer dollars, and leaving many unsuspecting students in high levels of debt. Students and workers bear the costs of reform. The intended goals of introducing more competition to offer students greater choice and to improve efficiency were not achieved. |
| Rogers, 2007 | To examine the impact of Australian government contracting for the provision of Job Network services on not-for-profit organisations, variously described as non-government, non-profit, community, and third sector organisations | The George Murray Essay 2006 | The conflict of not-for-profit service provider values with the values implied in policies has an impact on the ability of the organisations to represent the perceived interests of their members. Some organisations are disinclined to advocate against government policies for fear that their funding may be withdrawn. |
| Senate Legal and Constitutional Affairs Committee 2020 | To report on a Senate Inquiry into the impact of changes to service delivery models on the administration and running of Government programs | Report | Part of the inquiry reported on the impacts of outsourcing on constitutional and democratic responsibilities, the stability and quality of services, transparency, oversight, and the ‘public interest’ test. The committee suggested that the department fund and further explore options for developing an in-house solution that does not require the government to ostensibly ‘hand over’ control and management to private entities. |
| The McKell Institute, 2014 | To provide information concerning the health burden in Australia and whether the purported efficiency gains from privatisation offsets any potential decline in service quality. | Report | The risk of privatisation is not only damage to government budgets, patients, or hospital staff. It is also a significant risk to Australia’s universal healthcare system. |
| Tilley, 2020 | To trace key moments in the automation and privatisation of social security payments by a PPP with private company Indue Ltd. | Theoretical article | Under the guise of closing the digital divide, the ‘social harms’, and espoused objectives of the Cashless Debit Card administered by a private company are not being addressed. The things which ‘digital inclusion’ sought to counter, such as social stratification, inequality, exclusion and a loss of agency and decision-making, are being reproduced through heightened levels of control and surveillance. |
| Webster, 2001 | To present the results of a case study focusing on the implications of privatisation for regulating public utilities by using an example from the partially privatised Australian telecommunications industry. | Documents and database reporting | Privatisation of the telecommunications industry in Australia included regulation which ultimately led to disadvantaging rural communities. A major focus was on complying with on regulatory performance measures - or only doing what was measured. Other important elements of customer service were neglected, and rural customers suffered. |
| Young, 2008 | To explore a large Australian health contract; investigating reasons, processes, and outcomes. It investigates why the contract failed and the lessons to be learned from its subsequent awarding. | Case study | Together with cost savings, changes to work practices, and reduction in union power, the outsourcing contract involved problems with service quality, shared culture, relationships between contract and internal staff, and contract and staff management. There were reductions in trust and morale of both internal and contract staff. Inadequate contract speciﬁcations and subsequent under- pricing caused the contract termination, poor quality, and difﬁculties in contract management. |

Adapted from Younas & Maddigan (2019)
