# Supplementary material for: Privatisation of government services in Australia: what is known about health and equity impacts
Source: Global Health. 2024 Apr 16;20:32. doi: 10.1186/s12992-024-01036-w (PMC11020887; doi:10.1186/s12992-024-01036-w)
Supplement: Supplementary file 2 — Supplementary Material 2 [file 12992_2024_1036_MOESM2_ESM.docx]

**Appendix 2: Coding frame**

| **Nodes and sub-nodes** | **Context** |
| --- | --- |
| Benefitting corporations | To document references to the process of privatisation providing a clear benefit to corporations. |
| Equity | References relating to implications for equity from privatisation, eg impacts on marginalised groups |
| Health services | Documenting the privatisation of hospitals and other health services |
| Human services   - Vocational education - Early childhood - Prisons and correctional services - Employment - Aged care - Security - Detention centres - Child protection | Documenting the privatisation of human service functions formerly undertaken by governments. |
| Policy, planning and administration | Documenting the contracting out of public service policy and administrative functions to consultants. |
| Negative aspects of privatisation   - Cost - Inequity - Labour issues - Lack of accountability - Loss of government role and expertise - Service issues | Documenting any negative aspects of privatisation that can inform implications for health and health equity |
| Positive aspects of privatisation | Documenting any positive aspects of privatisation that can inform implications for health and health equity |
